# Direct Analysis and Quantification of Metaldehyde in Water using Reactive Paper Spray Mass Spectrometry

**DOI:** 10.1038/srep35643

**Published:** 2016-10-21

**Authors:** Simon Maher, Fred P. M. Jjunju, Deidre E. Damon, Hannah Gorton, Yosef S. Maher, Safaraz U. Syed, Ron M. A. Heeren, Iain S. Young, Stephen Taylor, Abraham K. Badu-Tawiah

**Affiliations:** 1 Department of Electrical Engineering & Electronics, University of Liverpool, Brownlow Hill, Liverpool, L69 3GJ, UK; 2 Department of Chemistry and Biochemistry, The Ohio State University, Columbus, OH, 43210, USA; 3 Northumbrian Water, Leat House, Pattinson Road, Washington, NE38 8LB, UK; 4 M4I, Maastricht Multimodal Molecular Imaging Institute, Maastricht University, Faculty of Health, Medicine and Life Sciences, Universiteitssingel 50, 6229 ER Maastricht, The Netherlands; 5 Institute of Integrative Biology, University of Liverpool, Crown Street, Liverpool, L69 7ZB, UK

## Abstract

Metaldehyde is extensively used worldwide as a contact and systemic molluscicide for controlling slugs and snails in a wide range of agricultural and horticultural crops. Contamination of surface waters due to run-off, coupled with its moderate solubility in water, has led to increased concentration of the pesticide in the environment. In this study, for the first time, rapid analysis (<~1 minute) of metaldehyde residues in water is demonstrated using paper spray mass spectrometry (PS-MS). The observed precursor molecular ions of metaldehyde were confirmed from tandem mass spectrometry (MS/MS) experiments by studying the fragmentation patterns produced via collision-induced dissociation. The signal intensity ratios of the most abundant MS/MS transitions for metaldehyde (177 → 149 for protonated ion) and atrazine (221 → 179) were found to be linear in the range 0.01 to 5 ng/mL. Metaldehyde residues were detectable in environmental water samples at low concentration (LOD < 0.1 ng/mL using reactive PS-MS), with a relative standard deviation <10% and an *R*^*2*^ value >0.99, without any pre-concentration/separation steps. This result is of particular importance for environmental monitoring and water quality analysis providing a potential means of rapid screening to ensure safe drinking water.

Detection and quantification of contaminants or pollutants in surface waters is of great importance to ensure safety of drinking water and for the aquatic environment^1^,^2^,^3^,^4^,^5^,^6^. Metaldehyde (CH_3_CHO)_4_ is a cyclic tetramer of acetaldehyde and is used extensively around the world as a molluscicide in agriculture for the control of slugs to protect crops. Large amounts of metaldehyde residues (from ‘slug pellets’) become mobilized, especially during periods of rainfall, seeping into reservoirs, rivers and groundwater, from which drinking water is sourced. Although metaldehyde has low toxicity, cases of metaldehyde poisoning and death in both humans and animals have been reported^6^,^7^,^8^. The United States Environmental Protection Agency (EPA) re-registered metaldehyde as a ‘restricted use pesticide’ and required risk-reduction measures to be adopted due to the potential short-term and long-term effects on wildelife^9^,^10^. The World Health Organization (WHO) classifies metaldehyde as a “moderately hazardous” pesticide (class II)^11^. In Europe, the European Commission has adopted a directive that restricts pesticides levels to 0.1 μg/L in drinking water^12^,^13^. Water companies and environmental agencies are under increasing pressure to routinely monitor levels of metaldehyde residues in water courses as part of their legal obligation^14^. As such there is an increasing need to develop effective analytical methods for detecting and quantifying metaldehyde in water samples at the source. In particular *in-situ* monitoring is required to ensure water management practices are based on empirical, up-to-date information which provides a better understanding of competing factors, risk and requirement.

Rapid analytical methods for *in-situ* analysis of metaldehyde in water, if available, would provide critical information on water quality for water companies and regulation bodies to manage exposures. Quantitative analysis of metaldehyde has been reported using various *ex-situ* methods based on solid-phase extraction^8^,^15^ followed by gas chromatography (GC) or high performance liquid chromatography (HPLC) with mass spectrometry (MS)^7^,^14^,^15^,^16^,^17^,^18^. However, each of these analytical methods involves extensive sample preparation including extraction, separation, and derivatization, resulting in increased cost and time of analysis. As will be demonstrated in this study, ambient ionization (AI) combined with tandem mass spectrometry (MS/MS) can overcome such limitations^19^,^20^,^21^,^22^.

AI is a form of ionization that is performed on unmodified samples in open air and the method is capable of providing almost instantaneous data while minimizing sample preparation^22^,^23^,^24^,^25^,^26^,^27^,^28^,^29^. Some of the most popular AI techniques include desorption electrospray ionization (DESI)^30^, extractive electrospray ionization (EESI)^31^,^32^,^33^,^34^,^35^,^36^, desorption atmospheric pressure chemical ionization (DAPCI)^37^,^38^,^39^, and direct analysis in real time (DART)^40^,^41^. AI-MS shows promise as an analytical tool for *in-situ* applications and has been demonstrated in a variety of fields where timely intervention is highly desirable such as: homeland security^23^, food safety^42^, pharmaceutical drug development^43^, and environmental monitoring^44^. There are several advantages to using *in-situ* AI methods capable of onsite analysis. The foremost advantage is the provision of data in real-time (or near real-time) at the point of interest allowing key management decisions to be taken in a timely manner. Subsidiary advantages relate to the chain of custody: by effectively taking the lab to the sample rather than the sample to the lab, the sample integrity is maintained and sampling/handling costs are significantly reduced.

The objective of the present study is to develop a new method for rapid detection and quantitative analysis of metaldehyde using AI-MS, based on paper spray (PS) ionization. PS-MS is a relatively new AI technique, first reported by Cooks, Ouyang & co-workers^45^ in 2010. PS has since been demonstrated for the analysis of a wide range of samples including bio-fluids^46^,^47^,^48^, bio-tissues^49^, protein complexes^50^, foodstuffs^51^,^52^,^53^, beverages^54^,^55^, bacteria^56^ and biocides^57^. The technique has undergone various developments such as, high throughput implementation^58^, application if carbon nanotube impregnated paper enabling low voltage application^59^, integration with solid phase extraction^60^ and printing hydrophobic wax barriers on to the paper substrate for extended solvent supply^61^. According to the authors’ knowledge, this study marks the first time that PS-MS has been utilized for the analysis of metaldehyde in water. The use of paper as a substrate material in analytical chemistry has been demonstrated for several decades and has many advantages such as: it has high surface area-to-volume ratio, it is readily available at low-cost, it wicks aqueous fluids, it is biodegradable and lightweight allowing for easy transportation and storage. In a typical PS experiment, a cellulose chromatographic paper is cut into equilateral triangles with ~5 mm sides using scissors and is wetted with a solvent. Charged droplets are emitted from the paper tip when a high DC voltage (±3–5 kV) is applied. Droplet emission occurs presumably via Taylor cone formation, which leads to analyte(s) ionization through electrospray like (and/or other unidentified) mechanisms^62^. Moreover, analysis by PS-MS requires little or no sample preparation and the entire full MS or MS/MS experiment can be completed within seconds (<1 minute). In comparison to other ambient ionization methods, PS integrates three analytical procedures: sample collection, separation, and ionization into a single experimental step making it more attractive for rapid and direct analysis of analyte(s) in complex mixtures. In addition, no nebulizer gases are required so the technique can be more readily used with portable MS in the field.

In the present study, experiments were carried out using a commercial benchtop ion trap mass spectrometer coupled with PS ionization (Fig. 1). Sample preparation was reduced to dissolving the model compounds (metaldehyde and paraldehyde) in methanol/water to form a stock solution (1000 ppm), that was serially diluted with water to the desired concentration before analysis, while raw environmental water samples (Abberton Raw & Chigwell Raw) were analyzed directly as supplied (from Northumbrian Water, UK) without any dilution. The results show that <0.1 ng mL^−1^ of metaldehyde in environmental water placed onto paper can be detected using a commercial benchtop mass spectrometer. The limit of detection (LOD) obtained was 0.05 ng mL^−1^ and below the permitted minimum EU levels for drinking water; good linearity (*R*^2^ = 0.9986) and accuracy (relative standard deviation ~7%) were also achieved. We further characterized the analyte(s) identity by analyzing the fragmentation patterns of metaldehyde in water using tandem mass spectrometry (MS/MS). We identified that the cyclic nature of metaldehyde can encourage the inclusion of different ions (H^+^, Na^+^ and NH_4_^+^) to enable the formation of corresponding metaldehyde ion types when analyzed using appropriate spray solvents. This capability was assessed in reactive paper spray experiments, offering more than an order of magnitude enhancement in detection limits. When collisionally activated, each ion type ([M + H]^+^, [M + Na]^+^, and [M + NH_4_]^+^) dissociated through unique pathways leading to the generation of distinctive product ions. These fragmentation patterns were fully characterized through MS/MS experiments.

## Results

### Analysis of metaldehyde using PS-MS

In this study we report the direct detection of residues of metaldehyde in water using PS-MS. Figure 2 shows the mass spectra of metaldehyde (*MW* 176) obtained in positive ion mode using paper spray ionization with methanol as the spray solvent. A dominant sodium adduct ion [M + Na]^+^ of metaldehyde at *m/z* 199 and a less intense ammonium adduct ion [M + NH_4_]^+^ at *m/z* 194 were observed (Fig. 2a). Insert (i) in Fig. 2a shows the isotopic distribution of the metaldehyde sodiated adduct [M + Na]^+^ at *m/z* 199. To confirm the identity of the molecular sodiated ion [M + Na]^+^ attributed to *m/z* 199, product ion MS/MS spectra were recorded using collision-induced dissociation (CID). The result from this experiment is shown in insert (iii), Fig. 2a, which indicates that, upon CID activation, the ion at *m/z* 199 yields a predominant fragment ion at *m/z* 67. This ion corresponds to sodiated acetaldehyde (*MW* 44) formed from the sequential loss of neutral dimer (*MW* 88) and monomer (*MW* 44) of acetaldehyde. Indeed, the intermediate fragment ion formed after the dissociation of the acetaldehyde dimer is observed at *m/z* 111, followed by the elimination of the monomer. A competing fragmentation pathway to the loss of the dimeric acetaldehyde was deemed to correspond to the elimination of a water (18 Da) molecule to give a less intense fragment ion peak at *m/z* 181. The less intense ammoniated molecular ion peak observed at *m/z* 194 was also confirmed via CID (see Supplementary Figure S1, Supplementary Information). Upon CID activation, the ion at *m/z* 194 yields a fragment through sequential loss of two water (18 Da) molecules, yielding intense product ions at *m/z* 176 and *m/z* 158 (major).

The sodiated molecular ion and fragmentation assignments were further investigated using deuterated metaldehyde-d_16_ (*MW* 192) as a model compound sample. Here too, a dominant sodiated molecular ion [M + Na]^+^ at *m/z* 215 was observed demonstrating that adduction with the Na^+^ ion was unaffected by isotopic substitution (Fig. 2b). Insert (ii) in Fig. 2b shows the isotopic distribution of the metaldehyde sodiated adduct [M + Na]^+^ at *m/z* 215. These sodium adducts were formed with relatively low internal energy as indicated by the absence of associated fragmentation observed in the full mass spectrum (Fig. 2). Insert (iv), Fig. 2b, shows the CID data for the intact metaldehyde-d_16_ sodiated molecular [M + Na]^+^ ion at *m/z* 215, which upon collisional activation dissociates yielding a more intense fragment ion at *m/z* 71 [CD_3_CDO + Na] via sequential elimination of dimeric (96 Da) and monomeric (48 Da) acetaldehyde-d_4_ without H/D scrambling. The stability and abundance of the precursor [M + Na]^+^ molecular ion from metaldehyde-d_16_ allowed multi-stage MS/MS/MS experiments to be performed and the result is as shown in insert (v), Fig. 2b, which unambiguously confirms the source of the *m/z* 71 product ion. Like metaldehyde, the deuterated metaldehyde-d_16_ species also formed adducts with ammonium ions at *m/z* 210.

The characterized sodiated [M + Na]^+^ molecular ions (*m/z* 199) provided a direct means to detect and quantify metaldehyde in water. This was accomplished by generating a calibration curve obtained using the collisonally activated fragment ion (*m/z* 71) intensity of the sodiated [M + Na]^+^ metaldehyde molecular ion at *m/z* 199 (Supplementary Figure S2, Supplementary Information). The limit of detection (LOD) was determined to be 2.69 ng/mL, which is above the EU regulated LOD value for metaldehyde in water (Table 1). The LOD was determined as the concentration that produces a signal more than three times greater than the standard deviation plus the mean value of the blank (in MS/MS mode). The sensitivity and selectivity of the PS-MS method can be enhanced by exploring chemical reactions that form stable adducts. To this end a more robust ionization mechanism of metaldehyde was developed in the form of reactive paper spray ionization.

### Reactive-PS-MS: Characterization and identification of protonated metaldehyde molecular ion species using formic acid

From the results observed in Fig. 2, it can be hypothesized that the sodium [Na]^+^ and ammonium [NH_4_]^+^ ions masked the protonation of metaldehyde. The introduction of reactive reagents in the PS spray solvent can improve the selective detection of metaldehyde in water; when used in combination with tandem MS, this approach can provide the confirmation needed to identify the presence of a particular substance in a complex mixture. This objective was achieved by adding acidified water (0.1% formic acid) to the methanol spray solvent MeOH:(H_2_O + 0.1% formic acid) (1:1, v/v). The addition of the acidified water greatly suppressed cationization (i.e. [Na]^+^ and [NH_4_]^+^ adduction) and aided protonation. The resultant PS-MS mass spectrum recorded when 5 μg of metaldehyde in 1 μL of deionized water was deposited on the paper substrate using MeOH:(H_2_O + 0.1% formic acid) (1:1, v/v) as the PS solvent is shown in Fig. 3. An intense, intact protonated molecular ion [M + H]^+^ of metaldehyde at *m/z* 177, including a major fragment ion at *m/z* 149, were observed in the single stage MS analysis (Fig. 3a). This fragment ion (*m/z* 149) appears to be formed from the elimination of ethylene (CH_2_ = CH_2_, *MW* 28 Da), even prior to collisional activation suggesting a ring opening/rearrangement process in the presence of formic acid.

This observation was further investigated in two experiments: (i) studies of gas-phase fragmentation patterns in tandem MS experiments and (ii) detection of paraldehyde under acidified spray solvent conditions. First, the structure of the protonated metaldehyde and its dissociation behavior were characterized after collisional activation. Insert (i) of Fig. 3a shows the product ion MS/MS mass spectra of the protonated metaldehyde. Unlike the sodiated molecular ion, which fragmented to give sodiated acetaldehyde (Fig. 2), the protonated metaldehyde species dissociates predominantly via the loss of CH_2_ = CH_2_ to yield a product ion peak at *m/z* 149. This fragmentation pathway indicates that the ion at *m/z* 149, observed in the full MS spectrum, is related to the metaldehyde protonated species and supports our suggestion that the sodiated molecular ions are formed with minimal internal energy deposition. This behavior was also observed using acidified spray solvent for the isotopically labelled metaldehyde-d_16_, yielding protonated molecular ions with a fragmentation pathway that also suggests a similar ring opening has occurred (see Supplementary Figure S3, Supplementary Information). In addition, CID of *m*/*z* 149 directly from the solution was compared with the fragmentation of gas-phase *m*/*z* 149 ion formed from MS_2_ of *m*/*z* 177 ion. The two spectra are similar where the mass of the main neutral loss is 28 Da providing an abundant ion peak at *m*/*z* 121 (Fig. S4, Supplementary Information). This result suggests that the *m*/*z* 149 ion generated in solution is the same in structure as the *m*/*z* 149 ion produced in gas-phase under CID. The second experiment to confirm the observed behavior of the protonated metaldehyde involved the use of paraldehyde, a cyclic trimer of acetaldehyde (metaldehyde being the corresponding tetramer). Figure 3b shows the positive ion mode mass spectrum of paraldehyde obtained when 5 μg of the sample was deposited on the paper substrate and sampled by using MeOH:(H_2_O + 0.1% formic acid) (1:1, v/v) as the spray solvent. A stable protonated molecular ion [M + H]^+^ of paraldehyde at *m/z* 133 was observed. The structure of the protonated paraldehyde species was confirmed from CID fragmentation patterns as shown in insert (ii) (Fig. 3b) where the molecular ion yields an intense fragment ion at *m/z* 89 owing to the neutral loss of acetaldehyde (*MW* 44 Da). Like metaldehyde, the fragment ion at *m/z* 89 was observed in the single stage MS experiment. Other signals were also observed such as at *m*/*z* 223 and *m*/*z* 164 in Fig. 3a,b respectively and their origins are not known.

The fragmentation pathway (*m/z* 177 → 149) for the protonated ion type was used to quantify metaldehyde in water (see Supplementary Figure S5, Supplementary Information). Using a commercial linear ion trap mass spectrometer, the LOD was determined to be 0.05 ng/mL. This quantitative analysis of metaldehyde in water was achieved from the metaldehyde calibration curve obtained with PS-MS using MeOH:(H_2_O + 0.1% formic acid) (1:1, v/v) as the PS solvent (Supplementary Figure S5). Following procedures established using LC-MS, deuterated atrazine-d_5_ (3 ppb, *m/z* 221 → 179) was chosen as the internal standard^17^,^63^. Monitoring the analyte-to-internal standard ratio (A/IS) as a function of analyte concentration yielded good linearity (*R*^2^ > 0.99), precision (RSD < 10%) and >fifty-fold decrease in the detection limit for metaldehyde in water compared with normal PS-MS, which utilized sodiated ions in the quantification process (Table 1).

### Direct metaldehyde quantitation in environmental water samples

Direct analysis of metaldehyde in a complex, raw, environmental water matrix using PS-MS was investigated without any sample preparation. The two water samples were collected directly from Abberton reservoir (Essex, UK) and Chigwell brook (Essex, UK) without any filtration except for large objects (>3 cm). Each sample had water turbidity levels of ~1.8 and ~0.79 NTU, total organic carbon ~6.5 and ~3.5 mg/L, and, pH ~8.35 and ~8.36, respectively. A volume of ~10 μL from each raw sample was deposited onto the paper substrate and analyzed using a commercial benchtop mass spectrometer in positive ion mode. Figure 4 shows the recorded mass spectrum for analysis of the raw water samples (Chigwell Raw and Abberton Raw supplied by Northumbrian Water Ltd.) using either MeOH or MeOH:(H_2_O + 0.1% formic acid) (1:1, v/v) as the PS spray solvent. Moderately intense protonated molecular ions of metaldehyde [M + H]^+^ at *m/z* 177 were observed in both water samples, and confirmed by MS/MS CID experiments (insert (i) & (ii) in Fig. 4) for the reactive experiment, which utilized an acidic spray solvent. Expectedly the presence of metaldehyde could not be confirmed in the same water samples when analyzed with the ‘normal PS-MS’ using methanol as the spray solvent.

## Discussion

The results obtained for the direct analysis of metaldehyde in water are apparent of the relatively lower sensitivity (higher LOD) of metaldehyde detection using the normal PS-MS method. The detection limit for sodiated metaldehyde is <3 ng/mL but is not suitable for *in situ* analysis due to the relatively high detection limit and the potential for salt concentration variations, which are likely to be encountered in the environment. Doping a reactive agent into the PS spray solvent enables reactions to occur at the sampling spot concurrently with mass spectra acquisition to aid both sensitivity and selectivity for target molecules present in complex mixtures. As such, experiments of this type (reactive PS-MS) were employed in this study to improve the detection of metaldehyde in water samples by more than an order of magnitude. Although the inclusion of a trace amount of acid is common practice for MS techniques to aid protonation; the addition of the acidified reagent in this case leads to ring opening (Fig. 5), hence the reactive nature of this process. We attribute the high sensitivity of the protonated ion type to the occurrence of only one major fragment ion in CID. The ability to form new ion type(s) from metaldehyde simply by adding reactive reagents (i.e. formic acid) into the PS spray solvent introduces an opportunity to differentiate metaldehyde from other potentially interfering ions having the same nominal mass. This advantage is particularly important for field metaldehyde analysis in which the selectivity of the paper spray method can be increased by studying the fragmentation patterns of sodiated (formed using neutral spray solvent) and protonated (formed using acidified spray solvent) metaldehyde species.

To understand this process (i.e., why [M+H]^+^ fragments differently than [M+Na]^+^) it was necessary to investigate the structure/nature of the suspected ring “opening” product formed in the presence of formic acid. As indicated in the results section, the elimination of 28 Da from metaldehyde was assigned to a loss of CH_2_=CH_2_ neutral species as illustrated in Fig. 5. This proposal is supported by the failure of acidified metaldehyde to react with hydroxylamine, both in solution and *in-situ* during reactive paper spray experiments. Product C is presumably formed via an internal proton hopping process and explains why both gas-phase CID and solution-phase rearrangements occur via a common ethylene loss.

PS-MS performed in the tandem mass spectrometry mode can reduce the effect of matrix ion suppression. For quantification purposes, a decision needs to be made as to which ions within the mixture should be subjected to collisional activation. In this respect, performing real time chemical reactions onsite will offer an efficient means to eliminate unrelated matrix ions. The generation of a charged product is expected to improve ionization efficiency of analyte(s) of interest in a complex mixture such as the protonation of metaldehyde in water. The combined reaction/ionization process is tested in this study for the analysis of metaldehyde (Fig. 6). As such both ionization efficiency and molecular selectivity can be improved by the addition of acidified reagents that can yield protonated molecular ions [M + H]^+^ for targeted analysis and quantification of metaldehyde in water samples (Fig. 3).

The ability to detect and characterize metaldehyde in raw water samples collected from natural water courses has been demonstrated. The concentration of metaldehyde in both water samples was cross-validated and confirmed to be <0.1 μg/L (the EU limit) using LC-MS. The detection limit obtained by PS-MS (Table 1) suggests that it could be suitable for the rapid detection of metaldehyde in raw water, although it registers higher values than those that can be obtained from GC- and LC-MS analytical methods^17^,^18^. With the capability of PS-MS to perform *in-situ* analyses of unmodified samples, the methodology described in this study shows promise for use in routine onsite investigative applications where regular monitoring or rapid screening is required. In this experiment ~10 μL from each sample was deposited on the paper substrate and analyzed using PS-MS. Figure 4 shows the recorded mass spectra for Abberton Raw (**a,b**) and Chigwell Raw (**c**,**d**). Moderately intense protonated molecular ions [M + H]^+^ of metaldehyde were observed for the reactive experiment and confirmed using MS/MS CID data as shown in Fig. 4, inserts (i) & (ii). The identification of the metaldehyde molecule in both water samples demonstrates the utility of the PS-MS method for direct, rapid screening with little or no sample preparation.

In summary, rapid and direct analysis of metaldehyde has been described using paper spray mass spectrometry. Sodiated [M + Na]^+^ and protonated [M + H]^+^ molecular ions produced under two different spray conditions (i.e. acidified MeOH:(H_2_O + 0.1% formic acid) (1:1, v/v) and normal MeOH PS solvents) were characterized in which [M+Na]^+^ species were identified to fragment through sequential losses of dimeric and monomeric acetaldehyde neutral species, whereas [M + H]^+^ dissociates via the elimination of ethylene. Quantitation of metaldehyde was achieved at low concentration (0.05 ng/mL for [M + H]^+^ and 2.69 ng/mL for [M + Na]^+^) in water using the reactive PS ionization method with acidified spray solvent. The MS/MS experiment provides a powerful means of qualitative analysis and confirmation of metaldehyde in water. The generation of different ion types in the specified spray conditions can offer an opportunity to readily discriminate (in the field) against other background ions with similar molecular weights since it is unlikely for a particular ion to fragment in a similar fashion as metaldehyde when using sodiated versus protonated parent ions in MS/MS. The demonstrated detection limit shows promise for the direct detection of metaldehyde in water at regulatory levels. Future work will involve further investigation/validation with untreated environmental water samples and pre-determined mock samples of varying water quality to determine potential ion suppression effects^64^ with a view to onsite *in-situ* analysis of metaldehyde and related environmental contaminants using a portable mass spectrometer. The objective is to translate the reactive methodology demonstrated with a commercial benchtop system, to a portable MS platform. Recent reports that couple ambient ionization methods, including PS, with portable mass spectrometers are promising^65^,^66^,^67^,^68^,^69^. In this respect, portable ion trap technology is often preferred as tandem analysis can be performed in time, without increase in instrument footprint. However with any portable system there is an inevitable trade-off between portability/field ruggedness and performance. For the purpose of onsite testing, in the context of water analysis, the trade-off is not as severe since the mass spectrometer can be confined to a vehicle without stringent restrictions on weight and power. The overarching goal is to achieve timely analysis, allowing near instant decisions to be made. Under current procedures, it can take up to 48 hours from sample collection until a determination is made. It is expected that such a portable setup will provide rapid analysis, being suitable for pre-screening and identifying local sources of pesticide contamination to inform operational decisions. Furthermore, due to the generic nature of MS, this methodology can be extended to other water pollutants that are of concern in the environment. As such, the results are significant beyond the analysis of metaldehyde discussed herein as they represent a means for rapid analysis of other environmental contaminants in water.

When coupled with a miniature mass spectrometer, the directness of the PS-MS experiment itself and the reactive alternative make for a potentially attractive on-site technique for water analysis and environmental monitoring. Other techniques such as the ‘leaf spray’ variant of the paper spray experiment^40^,^41^ can benefit by adopting this method for the analysis and determination of metaldehyde and other chemicals of concern on crops such as vegetables that may have been treated with pesticides and/or molluscicide^18^,^37^.

## Methods

### Chemicals, reagents and materials

HPLC grade methanol, formic acid and model compounds (i.e., metaldehyde and paraldehyde 99.9% purity) were purchased from Sigma-Aldrich (UK). The deuterium labeled standards, metaldehyde-d_16_ and atrazine-d_5_, were purchased from QMX laboratories (Essex, UK) while laboratory grade deionized water was purchased from Reagent Chemicals (Cheshire, UK). The chromatography paper used as the sample substrate was grade I Whatman filter paper (Whatman International Ltd., Maidstone, UK). The water samples (Abberton Raw & Chigwell Raw) were supplied by Northumbrian Water (Durham, UK).

### Sample preparation

Sample preparation was reduced to the dilution of model compounds to the desired concentrations whilst no sample preparation was performed for the raw water samples (Abberton Raw & Chigwell Raw). Each model compound was diluted in methanol/water (1:1 v/v) to 1000ppm stock solution and serially diluted in deionized water to the desired concentration so that appropriate ion abundances might be recorded; environmental water samples were used as supplied, from Northumbrian Water Ltd. (Durham, UK), without any modification or pre-concentration. From each solution, the 10 sample was deposited onto the filter paper surface, using a pipette and analyzed directly without any sample preparation. In all of the PS-MS experiments performed approximately 10 μL of pure methanol was used as the spray solvent (unless otherwise stated).

### PS-MS instrumentation

**Figure 1:**
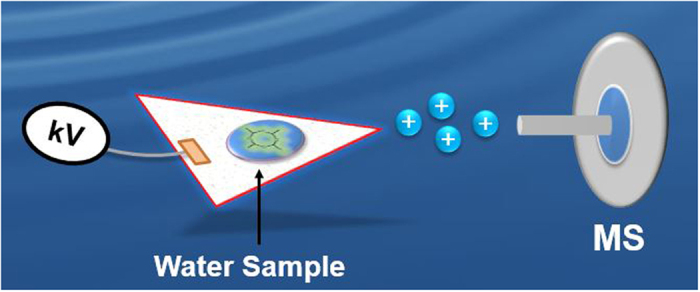
Schematic of the paper spray mass spectrometry experimental setup used for rapid detection of metaldehyde in water samples

**Figure 2:**
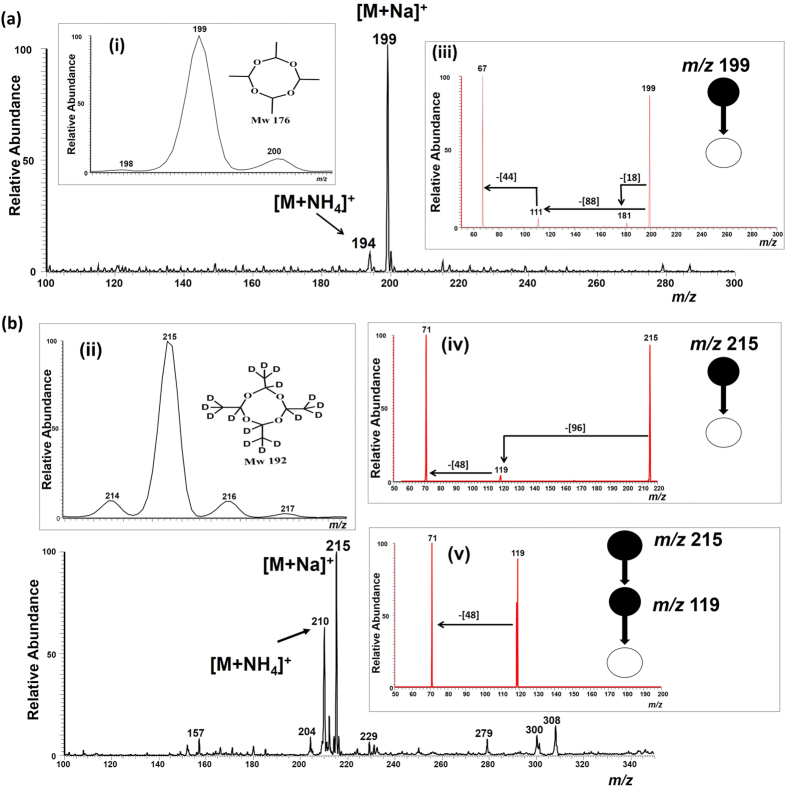
Positive ion mode paper spray mass spectrum of metaldehyde recorded using a bench-top ion trap mass spectrometer. 5 μg of the analyte in 1 μL of deionized water was spotted onto filter paper and ionized in air by application of a positive electric potential (3.5 kV) using methanol as the paper spray solvent. (**a**) The sodiated molecular ion [M + Na]^+^ peak of metaldehyde (*MW* 176) in deionized water produced the dominant ion signal intensity (*m/z* 199), and (**b**) Sodiated molecular ion [M + Na]^+^ of deuterated metaldehyde-d_16_ (*MW* 192) in deionized water produced the dominant ion peak (*m/z *215). Inserts (i–ii) show the isotopic distribution of the metaldehyde and metadehyde-d_16_ sodiated [M + Na]^+^ ion adducts at *m/z* 199 and 215 respectively. Note that in insert (ii) the relatively large signal intensity for *m/z* 214 is likely a consequence of D-H back-exchanges occurring in the ambient environment (and 99% isotopic enrichment). Inserts (iii–v) show the tandem MS CID data for the selected ions of metaldehyde and metadehyde-d_16_.

**Figure 3:**
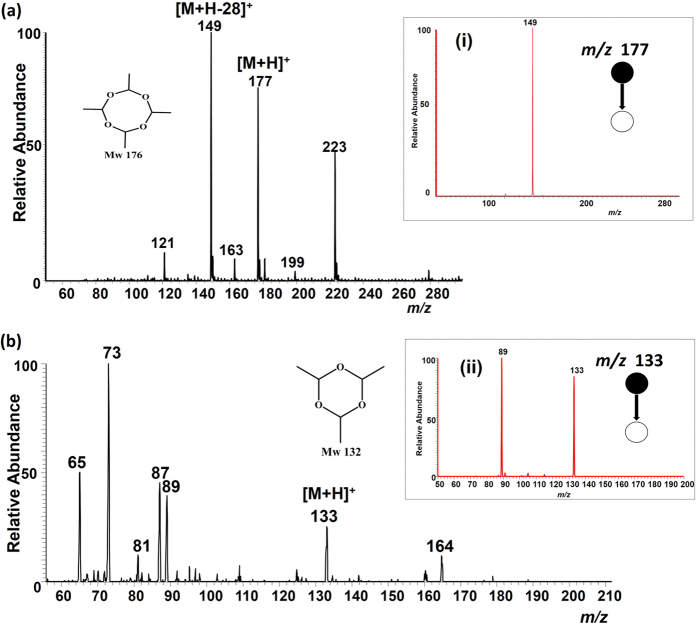
Positive ion mode paper spray mass spectrum using a bench-top ion trap mass spectrometer with MeOH:(H_2_O + 0.1% formic acid) (1:1, v/v) spray solvent application. 5 μg of the analyte in 1 μL of deionized water was spotted onto filter paper and ionized in air by application of a positive electric potential (3.5 kV); (**a**) metaldehyde and (**b**) paraldehyde. Tandem MS CID data for the *m/z* 177 and *m/z* 133 ions are shown in inserts (i) and (ii) respectively.

**Figure 4:**
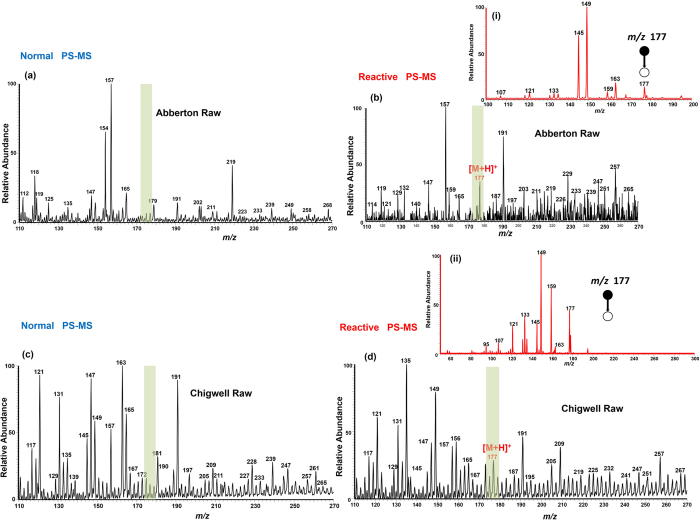
Positive ion mode paper spray mass spectra for rapid detection of metaldehyde in raw water samples (supplied by Northumbrian Water) whereby a volume of ~10 μL of the sample was deposited onto the paper substrate and ionized in the open environment by application of an electric potential of +3.5 kV. Abberton Raw was analyzed according to (**a**) the ‘normal PS-MS’ method and (**b**) with reactive PS-MS. Similarly for Chigwell Raw, ‘normal PS-MS’ analysis is shown in (**c**) and reactive PS-MS in (**d**). Inserts (i) & (ii) are the MS/MS CID mass spectra for the protonated metaldehyde ion at *m/z* 177 from each water sample analyzed using the reactive methodology.

**Figure 5:**
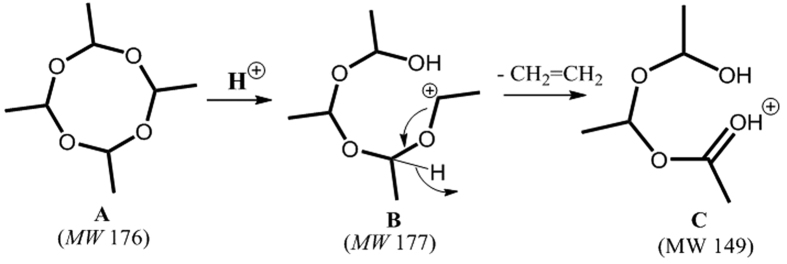
Proposed mechanism of acid catalyzed metaldehyde ring opening.

**Figure 6:**
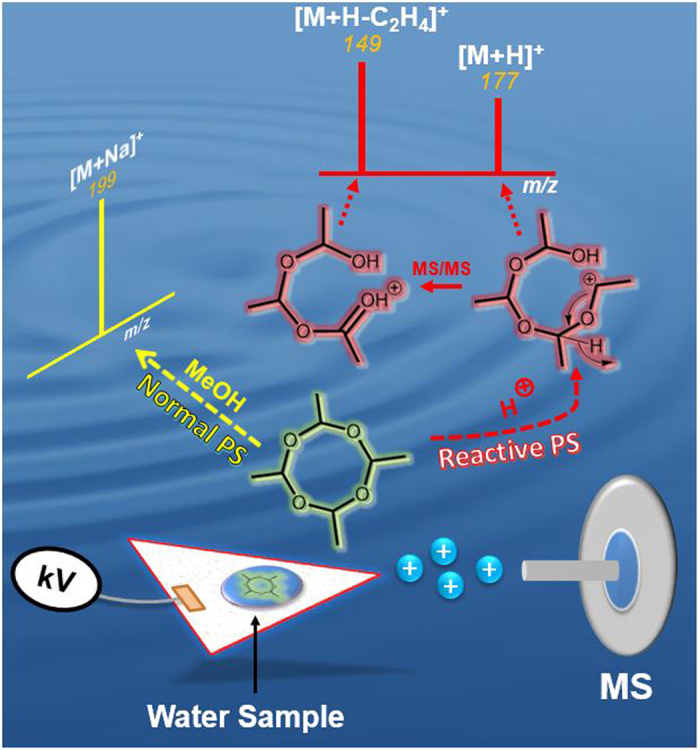
Illustrative diagram showing reactive and “normal” PS-MS analysis of metaldehyde generating different ion types.

All experiments were performed using a linear ion trap (LTQ) mass spectrometer (Thermo Fisher Scientific, San Jose, CA USA), tuned for optimum detection of the precursor ion of interest. The temperature of the MS capillary inlet was typically set at 200 °C. The tube lens voltage was set at 70 V and the capillary voltage maintained at 20 V in positive mode. The filter paper was cut manually into equilateral triangles with ~5 mm sides using scissors. The paper spray substrate was held by a copper clip so that the vertex was ~3 mm away from the inlet of the mass spectrometer. The sample solution was deposited onto the paper substrate followed by the application of an electric potential of 3.5 kV in positive ion mode. The mass spectrometer used is equipped with a differentially pumped atmospheric pressure inlet which acts to draw the PS ion plume into the vacuum system for mass analysis (Fig. 1).

It is important to note that in the paper spray experiments no carrier gas is required, instead a plume of ions is generated by the application of a potential on the paper with the sample and the spray solvent as shown in Fig. 1. Tandem mass spectrometry (MS/MS) was performed on the molecular ions of interest for structural elucidation allowing analyte identification using collision-induced dissociation (CID). An isolation window of 0.1–1.5 Th (mass/charge units) and normalized collision energy of 15–40% (manufacturers unit) were used.

## Additional Information

**How to cite this article**: Maher, S. *et al.* Direct Analysis and Quantification of Metaldehyde in Water using Reactive Paper Spray Mass Spectrometry. *Sci. Rep.* **6**, 35643; doi: 10.1038/srep35643 (2016).

## Supplementary Material

Supplementary Information

## Figures and Tables

**Table 1:** Analytical performance of PS-MS/MS for analysis of metaldehyde in water.

Figure of Merit	PS-MS/MS
LOD: [M + H]^+^ ion type	0.05 ng/mL
LOD: [M + Na]^+^ ion type	2.69 ng/mL
Estimated time of sample preparation	< ~ 60 seconds
*In-situ* analysis	Yes
