# Supplementary material for: Direct Analysis and Quantification of Metaldehyde in Water using Reactive Paper Spray Mass Spectrometry
Source: Sci Rep. 2016 Oct 21;6:35643. doi: 10.1038/srep35643 (PMC5073298; doi:10.1038/srep35643)
Supplement: Supplementary Information [file srep35643-s1.pdf]

Supplementary Information

Direct Analysis and Quantification of Metaldehyde in Water using Reactive Paper Spray Mass Spectrometry

Simon Maher[a], Fred P. M. Jjunju[a], Deidre E. Damon[c], Hannah Gorton[d], Yosef S. Maher[c], Safaraz U. Syed[e], Ron M. A. Heeren[e], Iain S. Young[b], Stephen Taylor[a] and Abraham K. Badu-Tawiah [c]**

[a]Department of Electrical Engineering & Electronics, University of Liverpool, Brownlow Hill, Liverpool, L69 3GJ, UK.

[b]Institute of Integrative Biology, University of Liverpool, Crown Street, Liverpool, L69 7ZB, UK.

[c]Department of Chemistry and Biochemistry, The Ohio State University, Columbus, OH, 43210, USA.

[d]Northumbrian Water, Leat House, Pattinson Road, Washington, NE38 8LB, UK.

[e]M4I, Maastricht Multimodal Molecular Imaging Institute, Maastricht University, Faculty of Health, Medicine and Life Sciences, Universiteitssingel 50, 6229 ER Maastricht, The Netherlands.

To whom correspondence should be addressed:

*Dr Simon Maher, Department of Electrical Engineering & Electronics, University of Liverpool, Brownlow Hill, Liverpool, L69 3GJ, UK, Telephone: +44 (0)151 794 9517. Fax: +44 (0)151 794 4540. E-mail: s.maher@liv.ac.uk

*Professor Abraham Badu-Tawiah, Department of Chemistry and Biochemistry, The Ohio State University, Columbus, OH 43210, USA. Telephone: +1 614 929 4276. Fax: +1 614 292-1685. E-mail: badu-tawiah.1@osu.edu

1. Characterization of Metaldehyde Detection using PS-MS

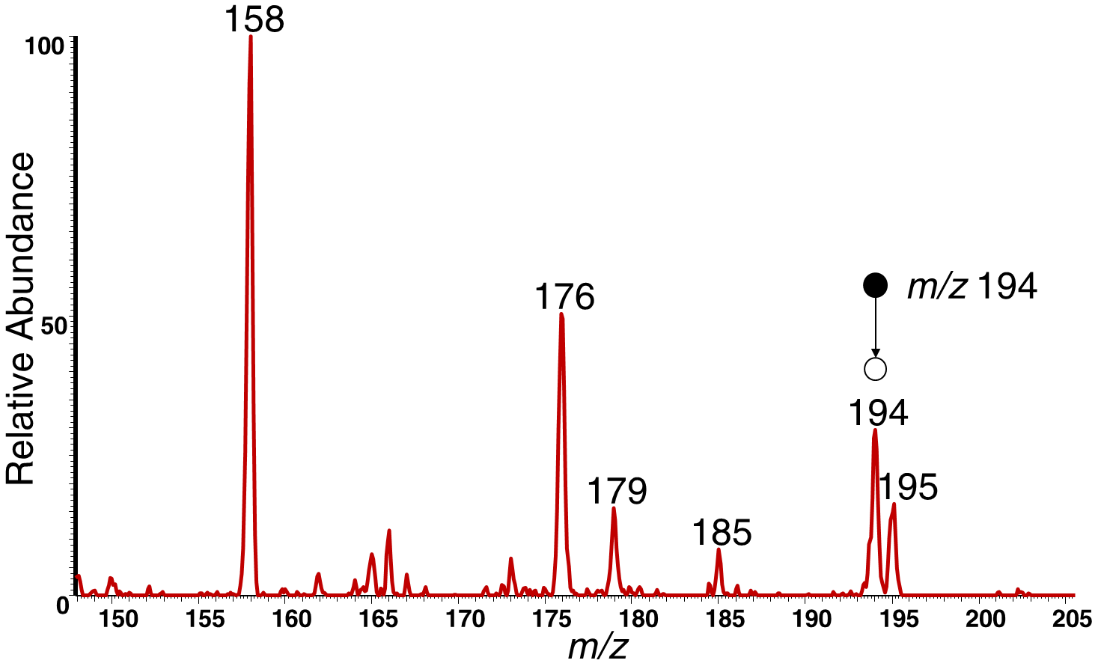

Supplementary Figure S1: Positive ion mode paper spray mass spectrum of metaldehyde recorded using a bench-top ion trap mass spectrometer. 5 µg of the analyte in 1 µL deionized water was spotted onto filter paper and ionized in air by application of a positive electric potential (3.5 kV) using methanol as the paper spray solvent. The figure shows the CID data for the precursor ion at *m/z* 194.

2. Neutral PS-MS calibration curves

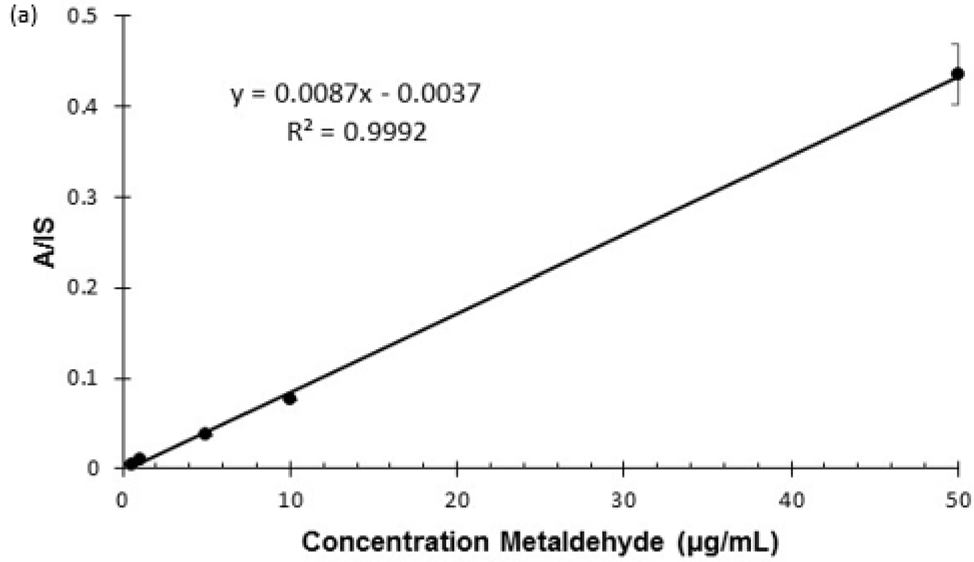

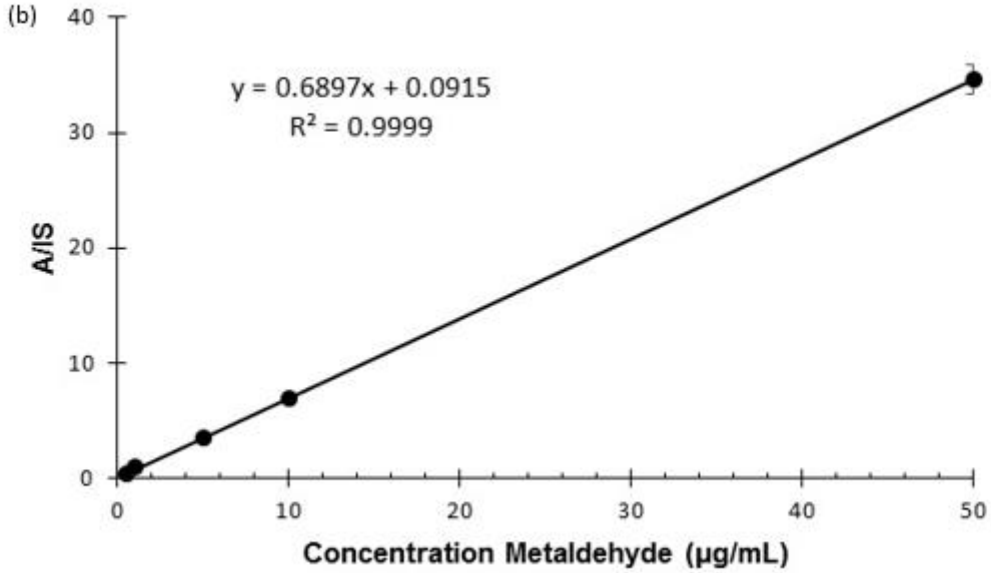

Supplementary Figure S2: Calibration curve for quantification of metaldehyde in water using PS-MS/MS when analysing (a) sodiated ion types and (b) ammoniated ion types produced in neutral MeOH spray solvent. Error bars indicate standard deviation from three replicates.

3. Characterization of acidified Metaldehyde-d16

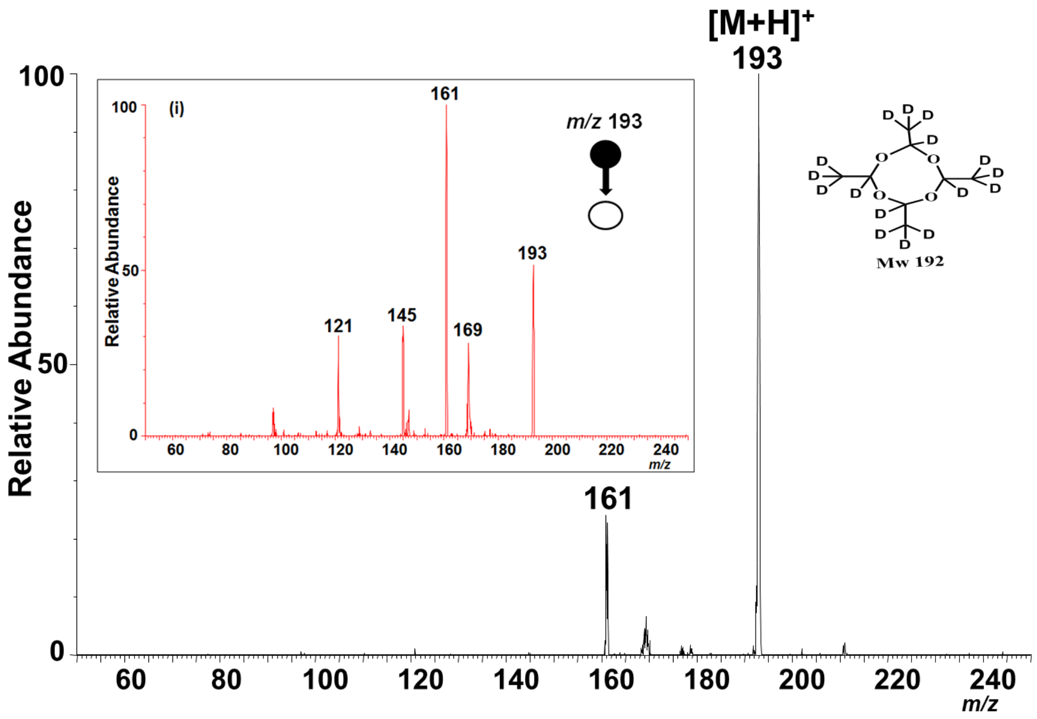

Supplementary Figure S3: Positive ion mode paper spray mass spectrum of metaldehyde-d16 using acidified spray solvent. Inset (i) shows CID data for the precursor ion at *m/z* 193.

4. Comparison of *m/z* 149 from solution and generated from gas phase fragmentation

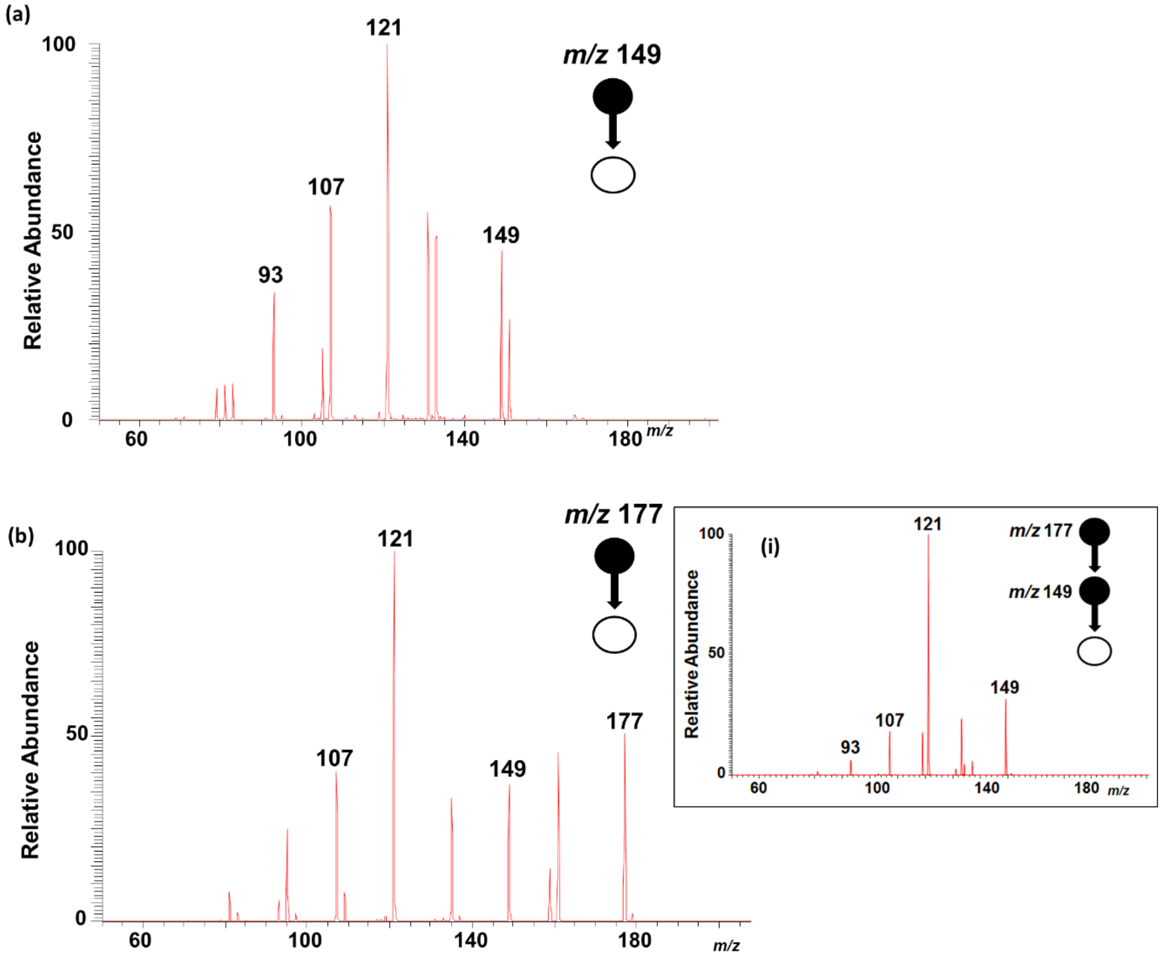

Supplementary Figure S4: Comparison of CID of *m/z* 149 formed (a) directly from the solution with (b) the fragmentation of gas-phase *m/z* 149 formed from the MS2 of *m/z* 177 (insert (i)).

5. Reactive PS-MS calibration curve

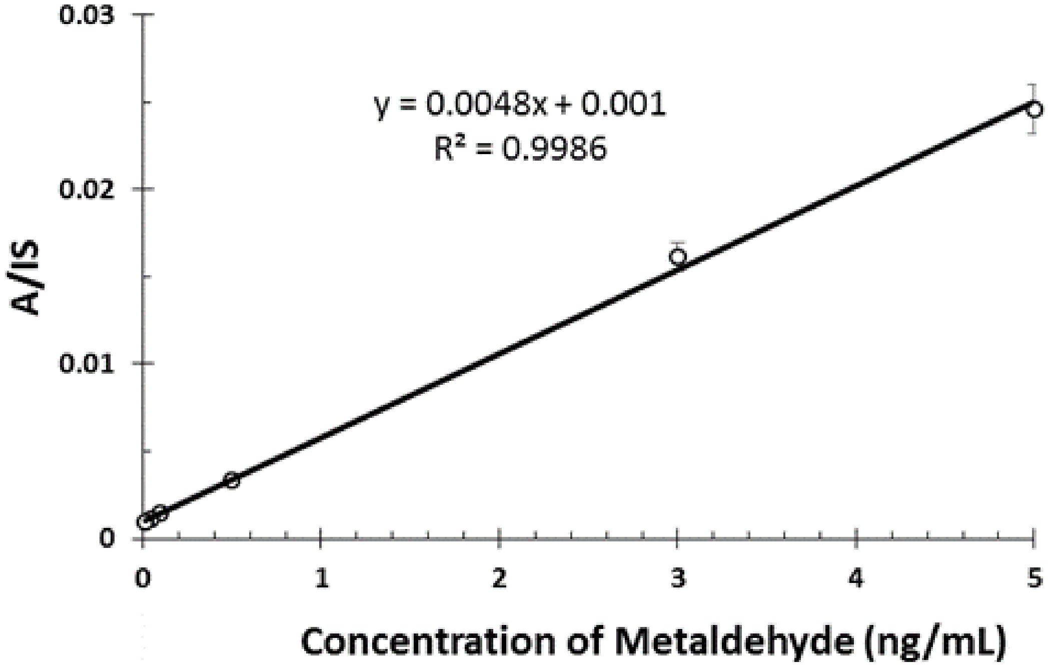

Supplementary Figure S5: Calibration curve for quantification of metaldehyde in water using PS-MS/MS when analyzing protonated ion types produced in acidified spray solvent. Error bars indicate standard deviation from three replicates.
